# Single-leg spica provides adequate stability after open reduction in developmental dysplasia of the hip

**DOI:** 10.1007/s00402-017-2845-1

**Published:** 2017-11-16

**Authors:** Nabil Alassaf

**Affiliations:** Department of Surgical Specialties, King Fahad Medical City, P. O. BOX 59046, Riyadh, 11525 Kingdom of Saudi Arabia

**Keywords:** Developmental dysplasia of the hip, Open reduction, Hip spica, Single leg, Redislocation, Children

## Abstract

**Introduction:**

The late detection of developmental dysplasia of the hip (DDH) will remain a major concern in some parts of the world until effective screening programs become available. With late diagnosis comes the need for open surgical reduction. Surgery is invariably followed by a period of immobilisation in a spica cast to prevent postoperative displacement. The goal of this study is to evaluate the effect of double-leg spica as compared to single-leg spica, on the risk of displacement after unilateral open reduction of the hip.

**Materials and methods:**

This was a retrospective review of DDH patients from 2012 to 2016 and younger than 4 years of age, who had unilateral anterior open reduction. Patients who had one of the following were excluded: neuromuscular diagnosis, the addition of K-wire, and simultaneous bilateral open reductions. Demographic data were collected along with related clinical and radiographic variables. A total of 128 patients (162 hips) met the inclusion criteria; 93 were in the double-leg spica group, and 69 were in the single-leg spica group.

**Results:**

The mean age was 25.4 ± 8.1 months and the mean follow-up was 18.6 ± 11.6 months. Baseline characteristics were balanced between the two groups. There were three events of redislocation in the double-leg spica group as compared to one redislocation in the single-leg spica group. The difference did not reach statistical significance (*p* = 0.637, risk ratio 1.317, CI 0.736–2.356). The difference in subsequent disruption of Shenton’s line and hip migration of more than 29% was (*p* = 0.395, risk ratio 1.411, CI 0.892–2.234) and (*p* = 0.087, risk ratio 0.67, CI 0.417–1.078), respectively. Three patients had a greenstick distal femur fracture after double-leg spica and one after single-leg spica.

**Conclusion:**

These data suggest that including the contralateral hip in the cast after open reduction is not essential as it does not seem to improve stability.

**Electronic supplementary material:**

The online version of this article (10.1007/s00402-017-2845-1) contains supplementary material, which is available to authorized users.

## Introduction

The term developmental dysplasia of the hip (DDH) spans several pathologies ranging from acetabular dysplasia without subluxation to frank dislocation. The incidence is as high as 6.6 in 1000 live births in populations that have no clinical and/or sonographic universal screening and approximately 2.2 in 1000 present late, mandating operative treatment [1]. After operative reduction of developmentally dislocated hips, the child is put in a hip spica cast. When the hip undergoes closed reduction, the opposite hip is invariably included in a frog-leg position [2]. In contrast, after open reduction with or without pelvic osteotomy, the hip is placed in a more neutral position with slight abduction. Currently, there is wide variation among practicing groups on whether to include the contralateral hip after unilateral procedures. Double-leg spica (DLS) or the one-half-leg spica casting is traditionally recommended and believed to better stabilize the hip. The single-leg spica (SLS), which is sometimes referred to as a walking cast, as a proportion of children walk in the cast, is used less frequently [3, 4]. Some practitioners extend the cast to include the toes of the affected extremity [5]. If delicate moulding is desired, plaster of Paris is used instead of lighter, stronger and waterproof fibreglass [6].

The superiority of SLS as compared to DLS with regard to burden on caregivers is unequivocally documented in previous publications [4, 5]. SLS spica was found to provide adequate stability in pediatric femoral fracture, but reports examining the adequacy of SLS after DDH reduction are lacking [3, 4, 7]. The goal of this study is to evaluate the effect of double-leg spica as compared to single-leg spica, on the risk of displacement after unilateral open reduction of the hip. The study hypothesis is that patients in the DLS group would have a lower probability of postreduction hip displacement.

## Materials and methods

### Selection of patients

An institutional review board approved this retrospective cohort study. Operating room medical records from October 2012 to June 2016 were screened. Patients who met the following inclusion criteria were included: a radiographic diagnosis of DDH, treatment by unilateral primary anterior open reduction, the absence of known teratologic or neurological disease, and aged less than 4 years old. Patients were excluded if hips had simultaneous bilateral open reduction, a stabilising Kirschner wire (K-wire), or were lost to follow-up during cast immobilisation.

### Baseline data

Electronic charts were reviewed for information related to age, sex, side, type of hip spica and severity of the disease using the International Hip Dysplasia Institute (IHDI) system [8]. Surgical notes were reviewed for evidence of open reduction and for whether pelvic or femoral osteotomies were done. Duration of cast immobilisation and duration of the follow-up were recorded from clinic visit notes.

### Outcome parameters

**Fig. 1:**
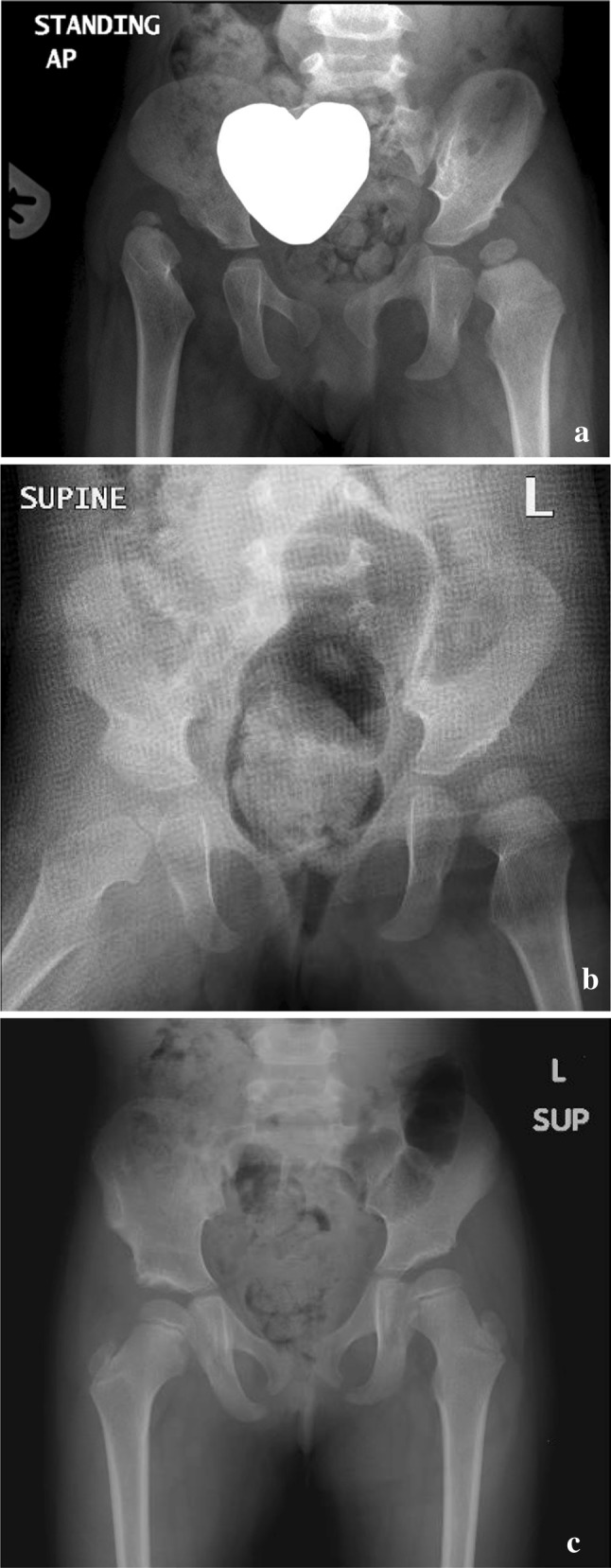
**a** A 2-year-old girl with right hip dislocation and dysplastic left hip. **b** Ten weeks after right hip open reduction and acetabuloplasty. **c** Follow-up radiograph postoperatively at 2 years, showing maintained right hip reduction and spontaneous improvement of left hip dysplasia

Final follow-up frontal pelvic digital radiographs (Centricity PACS; GE Medical Systems, Slough, United Kingdom) (Fig. 1) and the clinical notes, were examined for evidence of early redislocation producing uncoverage of the femoral head during cast immobilisation, late displacement, as defined by clearly interrupted Shenton’s line or a migration percentage (MP) of more than 29% as described by Riemers [9]. Information related to unplanned cast-related trips to the operating room, skin complications and insufficiency fractures secondary to immobilisation were collected.

### Treatment pathway

**Fig. 2:**
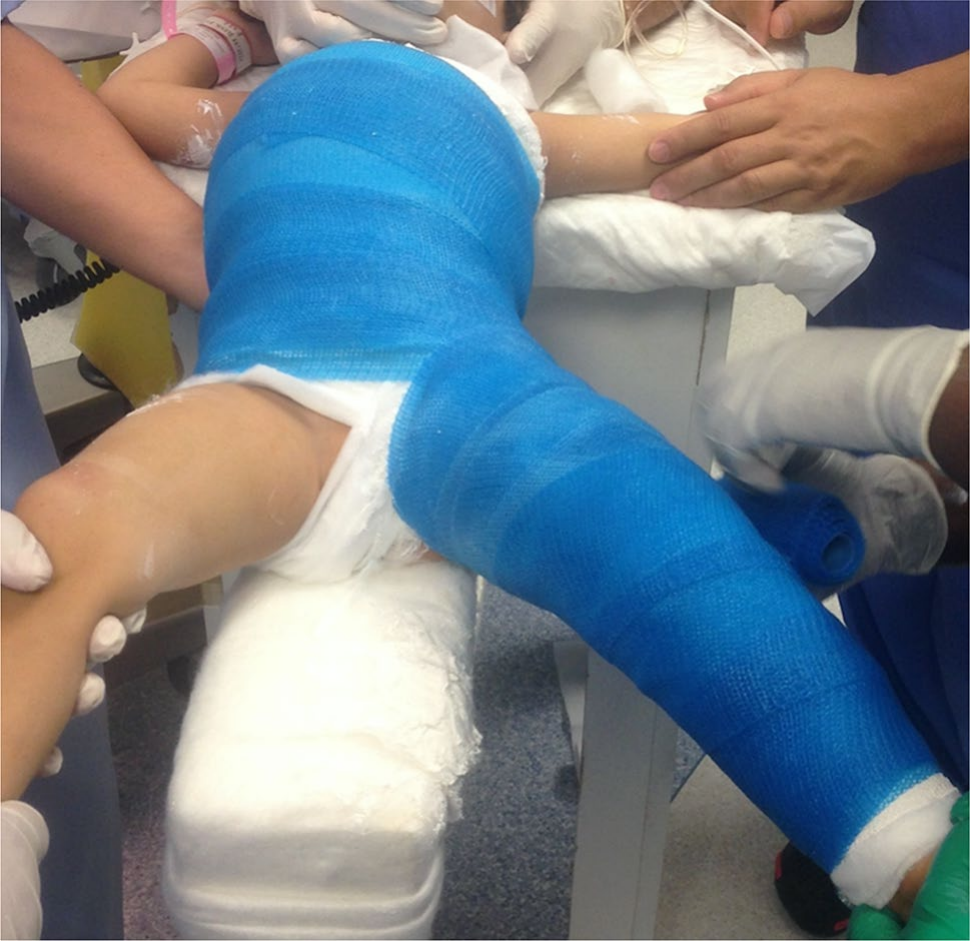
Clinical picture showing a single-leg cast application, after left hip open reduction

Capsular plication is done routinely after open reduction in all subjects. The type of hip spica was determined solely based on the surgeons’ routine practice using standard principles [10]. Three of the attending physicians with 3–4 years of experience in treating DDH, always used DLS with a connecting bar after open reduction of DDH and the upper part of the spica is removed in the clinic to free the hips for an additional 2–3 weeks, before complete discontinuation of the cast. The fourth attending physician who was in his first year of independent practice at the beginning of the study routinely used SLS after unilateral open reduction of the hip with augmentation at the hip area (Fig. 2). Casting was done immediately after the procedure and under general anaesthetic on a spica frame, followed by radiographic confirmation of concentric reduction before leaving the operating room and prior to hospital discharge. Fiberglass material was used for both groups with cotton padding.

### Statistical analysis

Statistical analyses were performed with R software, version 3.3.1 (R Foundation for Statistical Computing, Vienna, Austria). Normality of distribution and equality of variances were assessed graphically. Independent Student’s *t* test for parametric data and Mann–Whitney–Wilcoxon test for non-parametric data were applied when testing continuous variables. Chi-square test and Fisher’s exact tests were used to test proportions. A two-sided *p* value of 0.05 was considered significant.

## Results

**Fig. 3:**
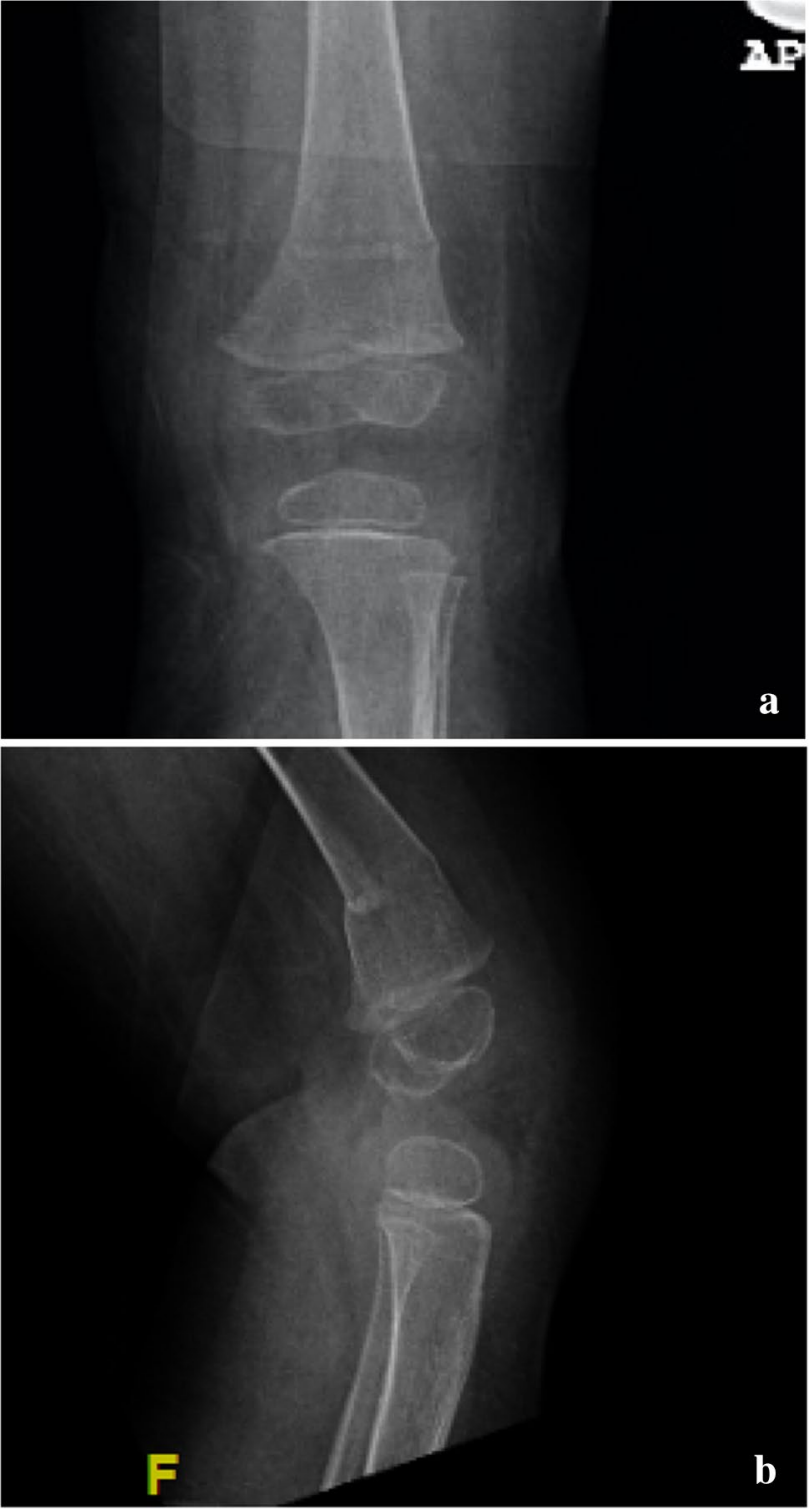
**a** Anteroposterior and **b** lateral femur radiographs showing osteoporotic fracture after casting, following ipsilateral open reduction

In total, 162 hips in 128 patients met the inclusion criteria, 104 girls and 24 boys. The mean age was 25.4 ± 8.1 months (range 11–48 months) and the mean follow-up duration was 18.6 ± 11.6 months (range 2–49 months). Seventy-one open reductions were in the right hip and 91 involved the left hip. The baseline parameters were not different between the two groups (Table 1). The early radiographic values during cast application and immediately after were not different as well as the risk of fractures secondary to disuse osteopenia (Table 2). These fractures were invariably undisplaced occurring in the ipsilateral distal femur (Fig. 3). Three patients in the DLS cast had unplanned trips to the operating room, one for manipulation for stiffness and two were for a dirty cast and two patients in the SLS group had an unscheduled cast change. There were no documented incidents of cast or skin breakage requiring special wound care in both study groups.

**Table 1:** Baseline characteristics

	Double-leg spica group (*n* = 93)	Single-leg spica group (*n* = 69)	*p* value
Age (months), mean ± SD (range)	25.2 ± 7.8 (13–48)	25.65 ± 8.56 (11–46)	0.717
Female gender	81 (87.1%)	51 (74.9%)	0.053
Right side	41 (44%)	30 (43.4%)	1
IHDI			
2	1 (1.1%)	1 (1.4%)	0.849
3	12 (12.9%)	7 (10.1%)	
4	80 (86%)	61 (88.4%)	
Pelvic osteotomy	89 (95.6%)	63 (91.3%)	0.327
Femoral shortening	11 (11.8%)	15 (21.7%)	0.138
Duration of casting (days), mean ± SD (range)	75.53 ± 25.05 (39–171)	72.78 ± 12.78 (35–120)	0.365
Duration of follow-up (months), mean ± SD (range)	18.85 ± 11.90 (2–48)	18.36 ± 11.19 (3–49)	0.794

*SD* standard deviation, *IHDI* International Hip Dysplasia Institute classification

**Table 2:** Results

	Double-leg spica group (*n* = 93)	Single-leg spica group (*n* = 69)	*p* value	Risk ratio	95% CI
Redislocation in the cast	3	1	0.637	1.317	0.736–2.356
Interrupted Shenton line	4	1	0.395	1.411	0.892–2.234
MP > 29%	11	16	0.087	0.671	0.417–1.078
Insufficiency fractures	3	1	0.637	1.317	0.736–2.356

*CI* confidence interval, *MP* migration percentage

## Discussion

SLS can potentially reduce cost and operative time. The impact of caring for the child with hip spica is substantial and has been quantified in the literature, and SLS has been shown to ameliorate the burden on the family [4, 5]. Hughes et al., examined the course of treatment on a 23 pediatric femoral fractures, where DLS was used, and they found a mean of 3 weeks of time off work by one of the working parents until adequate care arrangements could be made. The family’s greatest concern was transportation [11]. In a review of 45 patients who were put on SLS for pediatric femoral fractures, Epps et al., noted that 80% of the parents had missed an average of 2 weeks from work for the care of their child, and only 50% of children continued school or daycare [3].

Historically, postoperative casting protocols for DDH varied greatly in the literature. For example, Dega used DLS for 4 weeks, then the pelvic part is cut, and after a total of 6 weeks the entire cast was removed [12]. Pemberton used DLS for 2 months without any alteration during the casting period [13]. In addition, Salter recommended 6 weeks of single-leg casting followed by 4 weeks of bilateral toe-to-groin casts with an abduction bar [14]. In the present study, the redislocation rate was higher in the DLS group, but differences were not significant.

In contrast to DDH, there are a few reports on pediatric low-energy femoral fractures comparing SLS to DLS. Jaafar et al., retrospectively reviewed their experience in a cohort of pediatric femoral fractures in 59 subjects that had SLS and compared them to 35 patients in the DLS group over a period of 4 years. The SLS group had a lower rate of leg length discrepancy. Risk of loss of reduction was similar for both casting techniques [7]. In a randomised trial of pediatric femoral fractures, Leu et al., compared 24 patients in the SLS group with 28 patients in the DLS group. The authors found statistically significant more SLS adaptability to car seats and chairs as well as less time off work for the parents. For DLS the mean time off work by caregiver, was 19 and 10 days for the SLS [5]. Earlier, in a prospective cohort by Flynn et al., 19 patients were treated with SLS and 26 were treated by DLS. There was significantly less impact on the family reported score and the need for ambulance transportation with SLS [4].

Four patients (3.1%) had osteopenic fractures in this study, although this was not as common as fractures in chronically ill patients; caregivers may need to be aware of this risk. Fractures after casting for DDH are not extensively documented in the literature. Szalay and colleagues found one *z* score drop after 4–6 weeks of postoperative immobilisation or non-weight bearing in 15 orthopaedic patients. This was based on measurements from the distal femur, and most of the bone mineral density loss was in the cancellous bone and transitional regions [15].

Stability of the hip as defined by MP was not markedly different between the two groups. Based on the original work of Reimers, in patients with neuromotor hip disorders, MP was strongly correlated with the center edge angle of Wiberg. In the same landmark paper, 355 measurements were taken from urographic studies of children with no hip disease, and the highest recorded values were 29%. This cut-off value was used in the current study to indicated suboptimal femoral head coverage [9]. Careful longer-term follow-up is needed to ascertain satisfactory remodelling in hips showing higher values.

Five of the DDH patients required unscheduled trips to the operating room. In a study by DiFazio et al., 300 spica casts for pediatric femoral fractures were reviewed, and 24 patients required an unplanned trip to the operating room for cast change and 77 had skin complications [16]. None of the subjects in this study had wound care for skin ulcers, but our database is not sensitive to minor cast-related skin complications, mainly because a large proportion of the patients seek medical attention, and get cast trimming and padding at facilities near where they live.

This study has several limitations; it probably lacks adequate power to detect small differences in displacement rate between the two groups. It is somewhat assuring, however, that the redislocation rate was lower in the SLS group. Post hoc power analysis showed adequate power (> 80%) if a conventional margin of 10% was computed, but a 10% margin would be considered high by many. Stiffness was not studied as an outcome measure in this report for several reasons. These reasons were mostly due to the rarity of stiffness in children, the need for longer follow-up, inconsistent documentation and the poor reliability of current measurement tools [17].

Data presented here have a direct impact on DDH patients and their caregivers. Therefore, further research addressing the limitations of the current work is suggested. Moreover, the precise role of percutaneous pinning with casting in maintaining hip stability is unknown and the duration of immobilization remains empirical and could be elucidated in the future. In conclusion, SLS casting may be used after unilateral DDH open reduction procedures, without increasing the risk of early hip instability.

## Electronic supplementary material

Below is the link to the electronic supplementary material.

Supplementary material 1 (XLSX 26 KB)
